# Cell Death

**DOI:** 10.1155/2014/864062

**Published:** 2014-05-11

**Authors:** Claudia Giampietri, Alessio Paone, Alessio D'Alessio

**Affiliations:** ^1^DAHFMO-Section of Histology and Medical Embryology, Sapienza University of Rome, Via Scarpa 14, 00161 Rome, Italy; ^2^Department of Biochemical Sciences, Sapienza University of Rome, Piazzale Aldo Moro 5, 00185 Rome, Italy; ^3^Institute of Histology and Embryology, School of Medicine, Catholic University of the Sacred Heart, Largo Francesco Vito 1, 00168 Rome, Italy

Cell death is a crucial process involved in a variety of biological mechanisms controlling development, homeostasis, and immune regulation of multicellular organisms and its imbalance is associated with numerous pathologies. According to the literature, it is undeniable that the field of cell death research has been continuously growing and novel cell death modalities have been also described. Cell death can be classified according to morphological criteria identifying modalities such as apoptosis, necrosis, autophagy, or death associated with mitotic catastrophe. Additionally, cell death can be identified on the basis of biochemical mechanisms which include, for instance, the activation of different class of proteases (proteases, nucleases, and caspases) and according to the presence of specific cell surface molecules or the release of soluble mediators (immunogenic or nonimmunogenic cell death). The field of cell death is so complex that The Nomenclature Committee on Cell Death (NCCD) has recently proposed unified criteria which define the different types of cell death, while providing recommendations facilitating the communication among scientists involved in this field.

The main purpose of this special issue is to cover the field of cell death with themes focusing on pathways and mechanisms that specify active forms of cell death in health and disease. The topics therefore span apoptotic signaling networks (e.g., Bcl-2 family proteins, caspase control, novel molecular players of apoptotic control in immune regulation, and epigenetic regulation of apoptosis) and noncanonical cell death pathways, including necroptosis, pyroptosis, and autophagy, with a particular attention on the relationship between these mechanisms.

More specifically, M. E. Morrison et al. describe a novel role for the proapoptotic protein Bim in modulating cellular functions such as migration and extracellular matrix protein expression in retinal endothelial cells and pericytes. This study therefore provides additional regulatory mechanisms linking apoptosis control to vascular function.

M. Garg et al. focus on the regulation of apoptosis during the immune response. Their work highlights the role of the linker histone H1.2 trafficking in inducing T-effector lymphocytes apoptotic response after cytokine withdrawal. They demonstrate the well-controlled association between H1.2 and the proapoptotic mitochondrial resident Bak following metabolic stress.

M. N. Rossi and F. Antonangeli review our current knowledge of the role of long noncoding RNAs in apoptosis control. The authors highlight the altered expression pattern of specific lncRNAs in cancer cells when compared with normal cells and tissues. They also underline that overexpression or downregulation of different long noncoding RNAs in specific types of tumors may sensitize cancer cells to apoptotic stimuli. The authors discuss the new perspectives of using long noncoding RNAs as tumor biomarkers and as possible therapeutic targets.

M. D. King et al. show the novel role for the necroptosis inhibitor necrostatin-1 in limiting neurovascular injury in hemorrhagic injury models. This study emphasizes the potential clinical utility for necroptosis inhibitors as an adjunct therapy to reduce neurological injury after intracerebral hemorrhage.

C. Fabrizi et al., in the existing debate between pro- and antitumoral role of autophagy, draw attention on its significance in protecting cancer cells from death. The authors in fact demonstrate that pharmacological stimulation of autophagy may protect pheochromocytoma cells in stressful conditions such as high-density cultures and toxins exposure.

S. W. Ryter et al. give an exhaustive overview of the autophagic process. Furthermore, the authors discuss the impact of autophagy on the different cell death modalities (apoptosis, necroptosis, and pyroptosis). They underline the importance of strategies modulating autophagy for therapeutic interventions in diseases where cell death is deregulated.

A. Micera et al. investigate NGF and Reelin crosstalk in a genetic model of Reelin deprivation, namely, E-Reeler. Their results demonstrate that the loss of normal Reelin expression leads to early retinal impairment and loss of Rod Bipolar Cells in E-Reeler animals, damaging signaling from rod to ganglion cells. Altogether, their data propose Reelin as an important regulator in the retina which can be of great interest for studying mechanisms regulating retinal disorders.

C. Giampietri et al. give an overview of the molecular signalling of necroptosis. The authors focus on FLIP and IAP proteins that regulate the complex balance between apoptosis and necroptosis in particular in the immune system and in other physiological and pathological conditions. Finally, they discuss the deregulation of the necroptotic mechanisms in specific pathological conditions.

J.-R. Hong et al. reviewed the role of reactive oxygen species (ROS) in apoptosis and ROS induction by viral infection. They report how virus-mediated oxidative stress can participate to the etiopathology of several diseases by affecting immune cells and other tissues.

R. McFarland et al. employ the Lurcher animal model, which carries a mutated form of the glutamate receptor (GluR*δ*2L) in order to show that the over activation of the Na/K pump in response to the chronic Na^+^ leak is responsible for ATP consuming, leading to Purkinje cell death.

In summary, though cell death represents a major biological phenomenon, our understanding of its regulation in health and disease is far from being complete. The study of the multiple pathways driving to cell death has become increasingly complex and multidisciplinary, requiring expertise from all fields of the modern biology. Investigating the role of cell death and its regulation in the development of disease demands a constant update of our knowledge and the broadest interplay among both basic and clinical investigators. Therefore, better understanding of molecular signaling taking place during different types of cell death would not only foster the comprehension of disease pathogenesis but might be of crucial interest to develop new therapeutic strategies for treatment of cell death-related conditions. We believe that this special issue, due to the novel data presented and accurate reviews reported, will be appropriate for the broad readership interested in the new progress in the cell death field.

*Claudia Giampietri* *Claudia Giampietri* *Alessio Paone* *Alessio Paone* *Alessio D'Alessio* *Alessio D'Alessio*

